# Situating personality disorder within its maladaptive narrative identity ecology

**DOI:** 10.3389/fpsyt.2023.1117525

**Published:** 2023-03-09

**Authors:** Majse Lind

**Affiliations:** Department of Psychology, Aalborg University, Aalborg, Denmark

**Keywords:** narrative identity, disturbed narrative ecology, personality disorder, storying, multi-leveled storying, psychopathology

## Contemplating the role of narrative identity ecology in personality disorders

Personality disorders (PD) are characterized by rigid patterns of dysfunctional thinking, feeling, and behaving within most contexts and across various relationships (1–3). Categorical and, particularly, dimensional models of PD place identity at the very center of the disorder (4, 5). That is, within these models, the degree of disturbed identity reflects the very severity of PD (6) and, along with other central self-other features, acts as a driver of PD (7).

Following McAdams (8), identity takes the form of a story. Narrative identity constitutes the dynamic and evolving life story. Storying serves to scaffold a sense of continuity between time and place, by integrating diachronic and idiosyncratic actions into a meaningful whole, and supports a sense of purpose and direction as life unfolds (9). Importantly, narrative identity does not evolve in a vacuum as a private, intrapsychic process, but within a dynamic, psychosocial ecology (10)—a narrative ecology (11). Identity is not solo-authored but a co-authored process influenced by several higher-order levels (12, 13). Bronfenbrenner's ecosystem (10) has typically been used to illuminate how a person's narrative identity is reciprocally an influencer of, and influenced by, stories from the person's inner circle (Micro-Level), stories between microsystems (Meso-Level), stories from peripheral platforms such as one's neighborhood, local policy, and Mass Media (Exo-Level), and, finally, by the overarching cultural, political, and religious master narratives [Macro-Level, see also (11)]. Recently, the Chrono-Level was added to account for the aspect of time: environmental changes, life transitions, and historical events (10). As such, narrative identity evolves within a complex and dynamic narrative web of near and more distal storytelling. Indisputably, the ecological narrative network has been helpful to demonstrate conjoint interactions between what is considered to be the typical narrative identity and its typical environment. However, a comprehensive narrative ecology has not yet been presented, elucidating how the atypical or maladaptive narrative identity is mutually constructed within an atypical narrative ecology. This is particularly relevant in the context of mental illness in general and in PD specifically, as PD is repeatedly linked with disturbed narrative identity (14, 15). In two recently published reviews (15, 16), I display how individuals suffering from PD construct narrative identities differently from individuals without such pathology within at least three overarching narrative identity domains; structural elements (i.e., how the narrative is organized—the overall architecture), motivational/affective themes (i.e., what drives the narrator and their emotional tone), and autobiographical reasoning (i.e., the underlying, reflective process) (17). To summarize the most consistent findings, PD has been linked with more fragmented and idiosyncratic narratives that are lower on agency and communion fulfillment. The narratives are also predominantly negative (i.e., high on negative emotional tone and negative self-evaluations). Taken together, this work indicates PD is associated with several disturbances that traverse levels of the narrative ecology.

**Figure 1:**
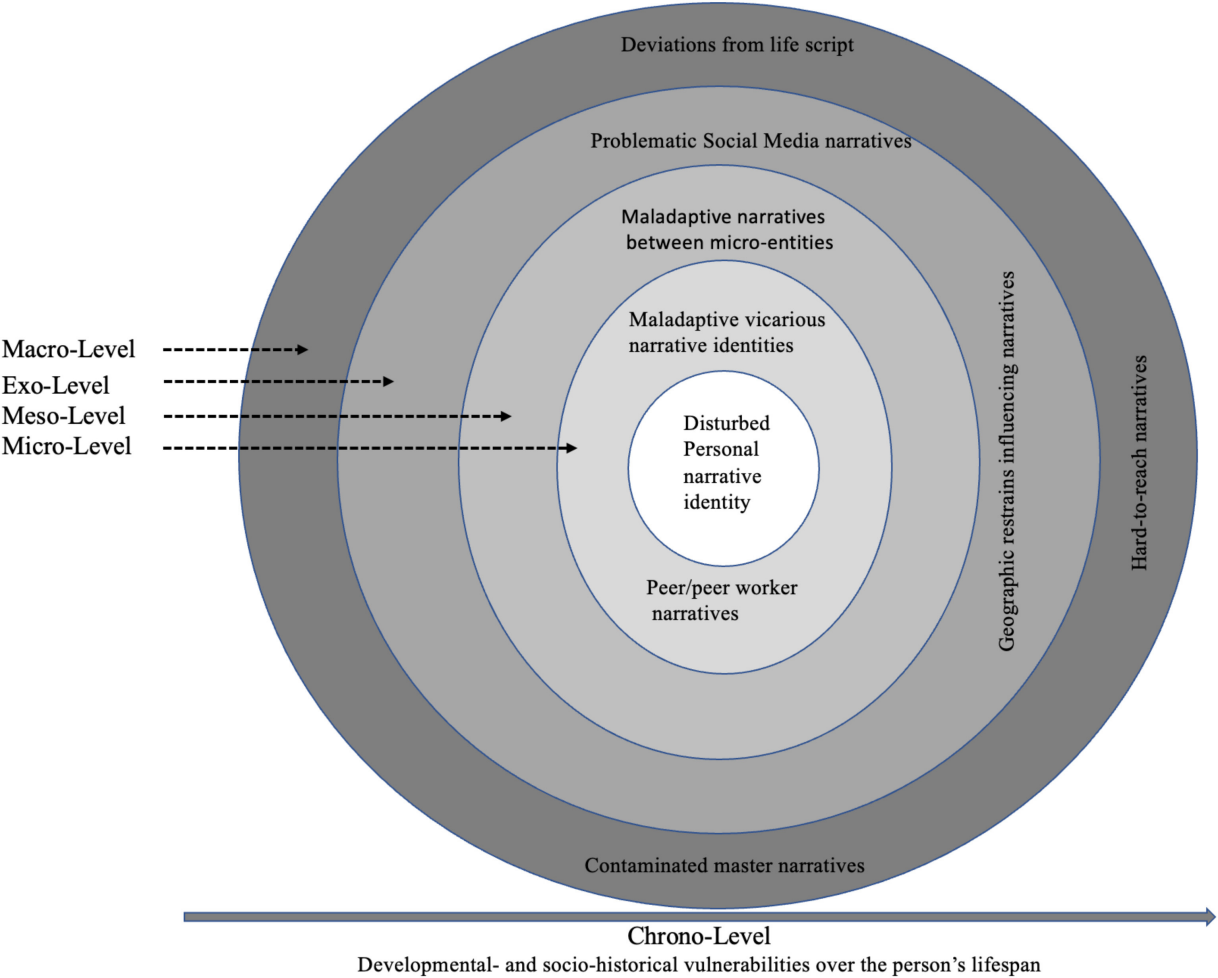
Visual illustration of the maladaptive narrative ecology in people manifesting PD.

Consequently, with this Opinion I aim to encourage researchers to shift away from studying disturbed narrative identity and PD in isolation and, instead, place the disturbance in relation to its rightful narrative milieu reaching a more nuanced, contextualized view of problematic storying in PD (see Figure 1).

## The Micro-Meso Level in PD

*If I ever try having a conversation with my mom, about anything, she will always start talking about her own upbringing, how mean her father was and how her mother never cared about her and how she never mattered to them [...] She has always felt worthless […]. According to her, this is the reason why she wasn't able to be a good mother to me. Because she didn't have any experiences with or have learned how to be a good mother […]*

Young woman with Borderline PD (18).

The vast majority of extant research on narrative identity in general (13) and in PD specifically (15), has focused on personal narrative identity in isolation. A few studies have started to build the narrative identity ecology from the Micro-Level in PD (see Figure 1). Inspired by Thomsen and Pillemer's (13) concept and work on vicarious life stories (i.e., the knowledge individuals have about other people's life stories), I showed that 30 outpatients with borderline PD crafted their parents' narrative identities in a similar way as they crafted their own narrative identities (18): lower on agency and communion fulfillment, and with more negative emotional tone and negative reasoning. The parents' narrative identities were also less complex than the personal narrative identities and with more self-other confusion. These studies represent a premature, though important, step toward outlining disturbances within the microsystem of the narrative ecology in PD. The studies have generated crucial questions for the future. For example, are the narrative similarities best explained by parents implicitly and/or explicitly teaching their children less adaptive, narrative strategies on how to understand themselves and their lives? Considering the above quote, the daughter continuously heard her mother craft a highly gloomy story about her childhood and uses the story to explain why she was not able to be a better mother herself. The story, induced with negative meaning-making and thwarted agency/communion, may seep through to how the daughter comes to understand herself and the type of narrative style to adopt. Problematic storytelling at the Micro-Level should be taken seriously because, once established, they may be harder to change in therapy compared to personal narrative identities in PD (19). Yet, more research is warranted to conclude anything definitively about change and stability in vicarious life stories. In addition to collecting vicarious stories from individuals with PD, future research should also collect personal life stories and vicarious stories directly from parents and additional close others (e.g., siblings, stepparents, and close friends). This would offer a more comprehensive picture of the type of narrative identities fluctuating within the inner circle, how they influence the person's storying, and how the person with PD may be sustaining the maladaptive narrative milieu. The individual's developmental stage [Chrono-Level; (10)] should also be considered in terms of when and what Micro-Level stories are most influential to the individual. For example, adolescence constitutes a particularly sensitive period for developing PD (20) and it is also within this period a disturbed narrative identity begins taking shape (21, 22). Thus, focusing on the maladaptive narrative ecology seems highly relevant in adolescents at risk of developing PD. Friends are considered key attachment figures in this developmental period (20) and peer-stories should therefore be considered at the very forefront at the Micro-Level. For that reason, it may also be particularly helpful to incorporate professional peer workers matched on age within the treatment of adolescents with PD since they might be more open to these stories [see also (20)]. Peers' stories can serve as alternative, optimistic, and empowering stories to the dominating stories of emotional tumult, interpersonal failures, and thwarted mastery they encounter from other PD patients at the hospital.

From the Meso-level, it is also important to consider links between the stories fluctuating between the Micro-Level entities (see Figure 1). For example, to what extent are stories about the person manifesting PD compatible between family, friends, school, and the psychiatric department? How are the entities communicating (if at all), and what stories may be told about each other? Researchers and clinicians should gather sufficient information about dominating storytelling within and across these entities and look for any deviations that may serve as a counter-narratives and potentials for growth. Despite this being a time- and resource demanding process it will possibly paint a richer and more holistic narrative of the person with PD across multiple contexts aligning with other, more recently developed PD treatments (e.g., AMBIT) (24).

## The Exo-Macro Level in PD

*I think it is really hard to accept that this is how things are (...) It's not fun when meeting new people, and they ask what you do, and you have to tell them that you are on sick leave. It is incredibly hard with how society works, and having a personality disorder has been the very worst, I think, because people are like “wow” […]. I am enrolled in education and really only need to finish my bachelor thesis and just started again recently, but after a week I had to go back on sick leave and that sucks.*

Young woman with borderline PD (18).

The young woman raises several, central societal concerns related to PD. The quote reveals a sense of self-stigmatization as feeling alienated from friends and acquaintances (25). Her description of people's “wow” reaction to the diagnosis further amplifies that PD, in her eyes, does not fit well with Macro-Level norms and values of the “typically narrated person” in the “typically narrated life” [see also, (23)]. Repeatedly, research has shown that individuals follow a culturally endorsed life script as a template when organizing their own narrative identity (26) and other people's narrative identities (13). Not surprisingly, a PD diagnosis is not part of the life script, and difficulties related to a PD diagnosis may hinder accomplishing some of the scripted events such as completing an education, as mentioned in the quote [see also (23)]. The painful deviations from the life script may be intensified by deviations from other powerful master narratives. In western cultures, redemption is one such master narrative, in which negative or challenging beginnings are eventually redeemed, for example by crafting ‘sickness to health' narratives (27, 28). A redemptive arc is seldomly related to the PD master narrative. On the contrary, clinicians have typically storied the diagnosis as “hard to reach,” a narrative that has only slowly started to change (29). Laypeople know very little about PD compared to other mental disorders (30), increasing the likelihood of false narratives about the disorder. Instead, the overarching master narrative related to PD seems to be one of contamination (31) with the story going from neutral or tolerable to bad (see Figure 1).

Challenges are also prevalent in the Exo-Level (see Figure 1): geographic differences often determine the treatment resources offered to people manifesting PD (20, 32). In psychiatric wards with fewer resources (e.g., less qualified staff, inadequate treatment possibilities), maladaptive narratives about the place and the patients are likely to grow faster and stronger. Social Media also plays a central role in the shaping of personal narratives (33). People with PD can more or less actively follow websites, forums, and Instagrammers posting stories about living with PD. The type of Social Media the person with PD may decide to follow will inevitably shape their personal narrative identity. Online spaces like Facebook provides a virtual, illness-related forum in which people deviating from the Macro-Level norms can find normality within subgroups (34). Hinson and Sword (34) describe how such distal, online communities can contribute to agency and authority and a sense of gaining a voice among equal co-authors. From a Chrono-Level perspective, researchers and clinicians should be particularly aware of the types of narratives gained from Social Media in young people with PD spending a significant amount of time within these channels. While Social Media narratives may, for some, contribute to agency and authority, they may also color narrative identity in maladaptive ways—for example, by finding community in this place, estrangement from society at large may grow stronger. Possibly, the new narrative identity may be used to explain away difficulties as something caused by the PD diagnosis and outside the individual's own control (i.e., diminished agency). Indeed, unlike some other mental illnesses or marginalized cultural identities, it may be more difficult to frame PD as a source of empowerment.

## Concluding remarks

In this Opinion, I have emphasized the importance of shifting away from studying narrative identity and PD as a private, intrapsychic process and, instead, place it within its maladaptive narrative ecology. I have flagged several types of problematic storytelling and offered suggestions on how to take the narrative milieu more into account. In future work, this, among others, means that narrative researchers should develop assessment that incorporates the different levels within the maladaptive narrative ecology. I am beyond excited to see this work unfold.

## Author contributions

The author confirms being the sole contributor of this work and has approved it for publication.
